# Conditional Gene Targeting: Dissecting the Cellular Mechanisms of Retinal Degenerations

**DOI:** 10.1155/2011/806783

**Published:** 2010-12-29

**Authors:** Yun-Zheng Le

**Affiliations:** ^1^Departments of Medicine, University of Oklahoma Health Sciences Center, 941 S. L. Young Boulevard, BSEB 302G, Oklahoma City, OK 73104, USA; ^2^Departments of Cell Biology, University of Oklahoma Health Sciences Center, 941 S. L. Young Boulevard, BSEB 302G, Oklahoma City, OK 73104, USA; ^3^Harold Hamm Oklahoma Diabetes Center, University of Oklahoma Health Sciences Center, 941 S. L. Young Boulevard, BSEB 302G, Oklahoma City, OK 73104, USA; ^4^Dean A. McGee Eye Institute, Oklahoma City, OK 73104, USA

## Abstract

Retinal neuron degeneration and survival are often regulated by the same trophic factors that are required for embryonic development and are usually expressed in multiple cell-types. Therefore, the conditional gene targeting approach is necessary to investigate the cell-specific function of widely expressed and developmentally regulated genes in retinal degeneration. The discussion in this review will be focused on the use of Cre/*lox*-based conditional gene targeting approach in mechanistic studies for retinal degeneration. In addition to the basic experimental designs, this article addresses various factors influencing the outcomes of conditional gene targeting studies, limitations of current technologies, availability of Cre-drive lines for various retinal cells, and issues related to the generation of Cre-expressing mice. Finally, this review will update the current status on the use of Cre/*lox*-based gene targeting approach in mechanistic studies for retinal degeneration, which includes rod photoreceptor survival under photo-oxidative stress and protein trafficking in photoreceptors.

## 1. Introduction

The use of gene targeting with homologous recombination in murine embryonic stem (ES) cells has led to many mechanistic insights about human diseases. However, global gene disruption has two major limitations that may prevent the identification of gene function in a target tissue or in adults. First, disruption of essential genes often causes embryonic or early postnatal lethality [1]. Second, disruption of a ubiquitously expressed gene may not yield mechanistic insights regarding the function of a protein of interest in a particular cell type [2, 3]. In these scenarios, temporal or/and spatial gene disruption is far more advantageous. The seminal work on the utilization of bacteriophage P1 site-specific recombination system in mammals by Dr. Brian Sauer and his coworkers [4, 5] established a firm foundation for the Cre/*lox*-based gene targeting, which is the most widely used conditional gene targeting approach to date. 

Cre recombinase is a 38 kDa protein and belongs to the integrase family of recombinases [6]. Biochemically Cre catalyzes site-specific DNA recombination, both intra- and intermolecularly, between the 34 base pair *loxP* sites [7]. Cre carries a eukaryotic nuclear targeting sequence [8] and is efficient in performing site-specific DNA recombination in mammals [9]. Therefore, Cre/*lox* system has become the primary choice for the site-specific DNA recombination-based manipulation of the mouse genome. Efficient Cre-mediated excision of DNA between directly repeated *loxP* sites has been widely used in gene activation and deletion of small or large segment of chromosomal DNA [9–11]. Cre-mediated recombination also permits the translocation of large DNA fragments on chromosomes [12] and integration (knock-in) or replacement of a gene or DNA segment [13–15]. Conditional gene knockout is by far the most widely used application of Cre-mediated site-specific recombination [16]. The use of this strategy in retinal degeneration studies will be the focus of this paper. In addition to the general strategy of Cre/*lox* gene targeting, this review will address various factors influencing the outcomes of conditional gene targeting studies, limitations of current technologies, availability of Cre-drive lines for various retinal cells, and issues related to the generation of Cre-drive lines. Finally, this paper will update the current status on the use of Cre/*lox*-based gene targeting approach in mechanistic studies for retinal degeneration, including the two most advanced areas, rod photoreceptor survival under photo-oxidative stress and protein trafficking in photoreceptors. 

## 2. Strategy in Experimental Design

### 2.1. Basic Scheme of Experimental Design

**Figure 1:**
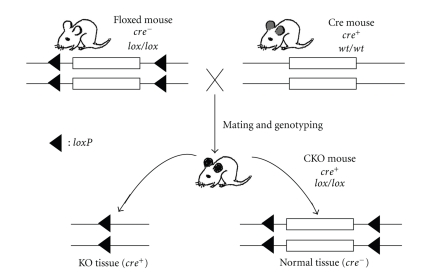
Schematic diagram of generating a conditional knockout (CKO) mouse from breeding a tissue-specific Cre mouse (top right) with a mouse carrying homozygous floxed gene (top left). A CKO mouse carrying a homozygous floxed gene and *cre* (either heterozygous or homozygous) is obtained by genotyping the F2 offspring. Tissue-specific Cre expression is shown as grey-eared (top right). Tissue-specific gene KO is diagramed as black-eared (bottom).

Cre/*lox* conditional gene targeting requires a mouse that has been pre-engineered with a *loxP*-flanked gene (or gene segment), generated with homologous recombination in murine ES cells (Figure 1). As the *loxP* sites are placed in introns, this engineered mouse is phenotypically wild type. A conditional gene knockout mouse is generated by breeding this mouse with a mouse that expresses Cre under the control of a tissue-specific promoter for two generations (Figure 1). In the conditional gene knockout mouse, the *loxP*-flanked gene is removed in a tissue-specific fashion. Only cells/tissues that express Cre carry the deleted gene, and thus they are phenotypically mutants (Figure 1). In this way, one can analyze the gene function in Cre-expressing tissues without affecting the gene expression in nontargeted tissues.

### 2.2. Considerations in Experimental Design

One concern regarding the use of conditional gene targeting *in vivo *is that the Cre-mediated excisive recombination is usually not 100 percent. Therefore, the effect of gene disruption may not be observed. It is important to understand that there is a fundamental difference between Cre-mediated gene disruption and conventional gene knockdown. As only four Cre molecules are required for a productive Cre-mediated recombination [7], Cre-mediated gene disruption occurs usually in an all-or-none fashion in a particular cell. A most likely scenario for a 20 percent efficiency of Cre-mediated recombination is that approximately 20 percent of targeted cells have 100 percent gene knockout. This is completely different from 20 percent gene knockdown in all cells. This characteristic has made Cre/*lox*-based gene targeting a useful approach in gene function analysis, even though it is rare that transgenic Cre mice express the recombinase in all targeted cells/tissues. Since most gene function studies are targeting the effect of gene inside the cells, a fraction of targeted cells with gene deletion could produce stable phenotypic changes in animals [44, 45]. However, in a scenario that no phenotypic change is observed in animals that have a small portion of targeted cells carrying Cre-mediated gene disruption, the interpretation of data needs to be cautious. 

Another misconception in designing conditional gene targeting studies is that a complete Cre-mediated excision is more desirable. This is not always true, particularly, in a situation that Cre may have toxic effect to the cells or phenotypic changes are too strong to be characterized. In a previous study, we intentionally used a rod-expressing Cre line with a lower efficiency of Cre-mediated recombination to avoid unnecessary complication derived from potential Cre toxicity in rods [44], as observed by others [21]. In a scenario that conditional gene targeting results in a massive or/and rapid phenotypic change that hampers the understanding of the biology and diseases, a lower level of Cre expression in targeted tissues/cells may produce a genetic mosaic that attenuates the development of pathological changes in animal models [46].

## 3. Cre-Drive Lines

### 3.1. Available Cre-Drive Lines

Although Cre can be exogenously delivered to a targeted tissue, it is usually expressed under the control of tissue/cell specific promoters. A critical factor for a successful conditional gene inactivation study is the availability of a suitable Cre-expressing drive line.  Table 1 includes a list of published Cre-expressing drive lines for various retinal cells. Since most retinal degeneration studies are related to the photoreceptors and RPE, all published rod-, cone-, and RPE-expressing Cre mouse lines are listed in Table 1. Retinal Müller glia is the major supporting cell and plays a critically role in maintaining structural and functional integrity in the retina under stress conditions. As most Cre-drive lines for Müller glia were usually developed for brain and Cre expression occurred outside ocular tissues in these mice, Table 1 only lists a few that either have been characterized more thoroughly or have been shown to be successful in conditional gene targeting in the retina [3, 47, 48]. Degeneration of retinal ganglion cells (GCs) is becoming a focused research area for their role in glaucoma and for the relevance to the safety of treating AMD patient with anti-VEGF strategies [49]. A number of characterized GC-expressing Cre-drive lines are thus listed in Table 1. While inner nuclear layer (INL) neurons are not often investigated for retinal degeneration, they are retinal neurons. The Cre-drive lines for INL neurons can be used for studies related to retinal neurobiology and are listed in Table 1. Finally, Cre-drive lines that are expressed in almost all retinal neurons are also listed in Table 1. It is worth noting that some of the listed Cre-expressing mouse lines were originally designed to trace cell lineage and had strong developmental Cre expression. These Cre lines may not be suitable for retinal degeneration studies. Although some promoters employed for Cre expression are useful in circumventing embryonic lethality, due to their ubiquitous expression they cannot be utilized to study a tissue/cell type-specific gene function.

### 3.2. Redundancy of Cre-Drive Lines

For most retinal cell-types, Table 1 lists more than one Cre-drive line. It is important to know that these seemly redundant Cre-drive lines are necessary. As most published Cre-drive lines derived from the same or similar promoters are not identical, it is ideal to have several usable Cre-drive lines for a particular cell-type due to the following considerations. First, a range of Cre expression levels provide choice to achieve a suitable degree of gene inactivation for a particular study. Second, variable ecotopic expression patterns between the Cre-expressing lines may produce unintended phenotypes that may be beneficial [24]. Third, transgenic *cre* is localized on one of the 20 chromosomes in mice. There is a 5 percent of possibility that *cre* may be residing on the same chromosome where a *loxP*-flanked gene is localized. Having more than one Cre-drive line for a targeted tissue/cell-type is likely to provide a choice for the successful generation of a conditional gene knockout mouse. Therefore, publishing a Cre-drive line for a particular cell-type with already established drive lines should be encouraged. Since there have not been many side-by-side studies comparing different Cre-drive lines as performed by Ivanova et al. recently [31], it is not possible to give an accurate account of the differences among Cre-drive lines that target a particular cell-type. This review only provides a roadmap about the available resources. To select the most desirable Cre-drive line, end users should perform side-by-side comparison, if necessary.

### 3.3. Types of Cre-Drive Lines

While the traditional transgenic approaches have proved to be useful for generating Cre-drive lines, the inherent problems associated with this approach [50] may cause variability in mutant phenotypes among animals. This variability sometimes may result in unintended expression pattern that may or may not be useful for other studies [24]. The use of knock-in or bacterial artificial chromosome based transgenic approaches is likely to produce Cre-drive lines with the expression patterns that more closely resemble the characteristics of the promoters. In addition, the variability in Cre expression among animals can be reduced using these transgenic approaches. For these reasons, the Cre-drive lines referenced in Table 1 also provide information on how these Cre-expressing mice were generated. It is important to keep in mind that a Cre-drive line generated with a knock-in approach may affect the expression of the native gene and careful phenotyping of Cre-expressing mice are necessary. 

Table 1 also includes information about whether Cre-expressing lines are generated using an inducible promoter system such as tetracycline- or tamoxifen-inducible systems [51, 52]. While inducible tissue-/cell-specific gene knockout approach is more advantageous, there are inherent problems associated with these systems, such as leakiness [53, 54]. Efficient delivery of inducing agents to the targeted retinal cells at the peak of promoter activity is the key to the success of inducible Cre expression. Although inducing gene expression in a tetracycline-inducible system with doxycycline for a short period of time may not be harmful to the retina [55], one should always keep in mind that tamoxifen may be toxic to the retina [56]. One distinctive advantage of using inducible systems is their ability to turn off/down the expression of Cre, which may be toxic to the targeted cells [19, 21].

### 3.4. Cre Toxicity

Cre is a DNA recombinase and may cause unintended chromosomal rearrangement at cryptic sites [57, 58]. Proper control of Cre expression is required for Cre-drive lines and a careful phenotypic analysis of Cre-drive lines is a prerequisite for conditional gene targeting. However, the Cre toxicity may not be the only contributing factor that caused retinal denegation in Cre-expressing rod-specific Cre mice [19, 21]. As expression of human rhodorpsin-GFP fusion, a nontoxic protein, also caused progressive rod photoreceptor degeneration [59], it is likely that a high level of expression of an exogenous protein may be toxic to the host protein transcription/translation/maturation system in rods.

### 3.5. New Cre-Drive Lines

For the past decade or so, many laboratories have contributed considerable effort in establishing various Cre-drive lines. While Cre-expressing mice have been used successfully in conditional gene targeting, there are not sufficient Cre-drive lines, even for the most advanced field, photoreceptor biology. Due to a high level of Cre expression causes rod degeneration, it would be ideal to have at least one inducible Cre-drive line for rods. As there are at least fifty types of retinal neurons, the current list (Table 1) is far from completion. However, for most retinal cell-types, a major shortcoming of most currently available Cre-drive lines is a lack of temporal or spatial specificities and desired efficiencies. Significant improvement in this area is needed. At present, a major challenge for Cre/*lox*-based conditional gene targeting is the difficulties to obtain Cre-drive lines with desired tissue-specificities. A lack of “ideal” promoters is the major reason. Therefore, it is worthwhile to invest some effort on studying the expression pattern of potential promoters that drive Cre expression before making a mouse.

## 4. Dissecting Cellular Mechanisms of Retinal Degeneration

### 4.1. Photoreceptor Survival under Photo-Oxidative Stress

A major focus in retinal denegation is to reveal the mechanisms of photoreceptor survival. As many of the survival factors are essential for development, global disruption of these essential genes often causes embryonic lethality. Using Cre/*lox*-based conditional gene targeting approach, Haruta et al. demonstrated that Rac1, a component of NADPH oxidase that produces reactive oxygen species, was required for the rod photoreceptor protection from photo-oxidative stress [60]. To determine photoreceptor survival mechanisms under photo-oxidative stress, Ueki et al. used rod-specific gp130 knockout mice and showed that preconditioning of mice with a sublethal photo-oxidative stress activated an autonomous protective mechanism in rods through gp130, an IL6 cytokine receptor, and, its downstream target STAT3 [61]. To determine further whether Müller cells, major retinal supporting cells often played a role in photoreceptor protection by releasing survival factors, were involved in this process, they demonstrated that gp130 activation in Müller cells had no additional effect for rod survival under photo-oxidative stress [47]. While this study demonstrates the neuroprotective role of gp130-STAT3 pathway in the rod photoreceptors under the chronic photo-oxidative stress, another series of studies showed that the PI-3 kinase/AKT pathway could protect rod photoreceptors under the acute photo-oxidative stress. Using a conditional gene knockout approach, Rajala et al. showed that insulin receptor, a PI-3 kinase upstream regulator, had a protective effect to rod photoreceptors under the acute photo-oxidative stress [62]. In another study using a conventional gene targeting approach, disruption of AKT2, a PI-3 kinase downstream target, accelerated the acute photo-oxidative stress-induced rod photoreceptor degeneration [63]. Finally, Zheng et al. demonstrated that BCL-xl, a downstream target of AKT, was a rod survival factor under acute photo-oxidative stress [44]. These studies clearly mapped the significance of PI-3 kinase/AKT pathway in stress-induced rod photoreceptor survival *in vivo.*

### 4.2. Protein Trafficking and Photoreceptor Degeneration

Kinesin-II is a molecular motor localized to the inner segment, connecting cilium, and axoneme of mammalian photoreceptors. The involvement of kinesin-II in protein trafficking through the mammalian photoreceptor cilium was initially probed with Cre/*lox*-based conditional gene targeting. Loss of kinesin-II in rods caused significant accumulations of opsin, arrestin, and membrane proteins within the photoreceptor inner segment, which ultimately led to the death of photoreceptors, a phenotype that is commonly observed in retinitis pigmentosa [20]. Further experiments also suggested that ectopic accumulation of opsin was a primary result of rod-specific kinesin-II deletion [21]. Using a conditional gene targeting approach, Avasthi et al. recently demonstrated that heterotrimeric kinesin-II acted as a molecular motor for proper trafficking of membrane proteins within the cone photoreceptors [64]. These conditional gene targeting studies established an unequivocal role of kinesin-II as a molecular motor that facilitates protein membrane trafficking in the photoreceptors.

### 4.3. Conditional Gene Targeting in the RPE

RPE is the gatekeeper of the retina and plays a pivotal role in the maintenance of retinal neurons. Abnormal RPE function is associated with both the wet and dry-forms of age-related macular denegation (AMD) (for review see [65, 66]). Although the pathogenic mechanisms for dry-AMD is unclear, clinical evidence suggests that photoreceptor degeneration is a consequence of impaired RPE functions [67, 68]. RPE-specific gene targeting will be a powerful approach for functional analysis of the RPE-expressed genes in the pathogenesis of dry-AMD. Whereas the use of conditional gene targeting in the PRE is still at its infancy, investigating the role of vascular endothelial growth factor (VEGF or VEGF-A), a potent angiogenic factor whose polymorphisms are associated with AMD [69, 70], in choroidal vascular development has yield some information related to the relationship between the RPE-derived VEGF and choroidal vasculature [2, 71]. As abnormal choroidal vasculature is clearly associated with both the dry- and wet-AMD [72–75], the genetic systems established in these studies may have some utility for AMD research. While the conditional gene targeting approach has yet to reach its full potential in AMD research, Lewin et al. recently demonstrated that disruption of mitochondrial manganese superoxide dismutase (SOD) in the RPE produced a geographic atrophy-like phenotypes in mice [76]. Here again, tissue/celltype-specific disruption of widely expressed genes, such as VEGF and SOD, circumvents the interference of nontargeting tissues/cells and is likely a direction for generating animal models used for mechanistic, diagnostic, and therapeutic investigations in the years to come.

## 5. Concluding Remarks

Remarkable progress has been made since the publication of the first study on the retinal denegation using a conditional gene targeting approach a decade ago [20]. It is also important to realize that, except in protein trafficking and photoreceptor survival, progress in other areas of retinal biology is not keeping the pace. At present, cellular mechanisms of many trophic factors and their signaling pathways in the retina remains unclear. Although the RPE and Müller cells are two major retinal supporting cell-types, the post-developmental functions of RPE and retinal Müller cell-derived trophic factors and their signaling mechanisms have remained largely uninvestigated. Substantial effort is necessary to establish a framework for cellular mechanisms of inherited retinal degeneration, AMD, and diabetes-induced retinal neuron degeneration. Many of these investigations will require the use of conditional gene targeting approach. With the improved Cre-drive lines and effort in investigating cell-specific function of trophic factors and their signaling, significant progress in our understanding of retinal degeneration will be achieved in the near future. Ultimately, these findings will help to design therapeutic approaches for the treatment of the retinal degenerative diseases.

## Figures and Tables

**Table 1:** Published potentially useful Cre-drive lines in designing studies related to retinal degeneration.

Major targeted cells	Minor/other expression	Promoter	References
Photoreceptors			
M- and S-cone	Not reported	*hRgp*	[17]
M-cone	Not reported	*mMo*	[18]
S-cone	Not reported	*mSo*	[18]
Rod	Rod bipolar	*mRho*	[19]
Rod	Not reported	*Irbp*	[20]
Rod	Not reported	*hRho*	[21, 22]
RPE			
*RPE	Optic nerve	*hVmd2*	[23]
*RPE	Müller cells/optic nerve/INL	*hVmd2*	[24]
RPE	Pigmented cells	*Dct*	[25]
RPE	Neural retina	*Trp1*	[26]
RPE	Lens/neural retina	Modified *αA-crystallin *	[27]
Müller glia			
^#^Müller cells	GC and ONL	*Pdgfra*	[28]
*Müller cells	INL	*hVmd2*	[24, 29]
^!#^Müller cells	Brain	*Glast*	[30]
Müller cells	INL/Brain	*Thy1*	[31, 32]
Müller cells	Brain	*Foxg1*	[31]
Ganglion cells			
GC	Brain	*Grik4*	[31]
Melanopsin-expressing GC	Not reported	*Opn4*	[33]
^$^ *GC *	Amacrine and horizontal cells	* Math5*	[34]
*GC/n*eural retina	Brain	*Thy1.2*	[35]
GC/Amacrine cells	Brain	*Chat-*(BAC transgenic)	[31, 36]
Inner nuclear layer neurons			
^$^Amacrine cells	Not reported	*Chat-*(knockin-Jackson Lab)	[31]
Bipolar cells	photoreceptor/Brain	*Pcp2*	[37]
^#^Rod bipolar cells	Brain	*Pcp2*	[38]
^$^Amacrine and horizontal cells	Not reported	*Ptf1a*	[39]
Neural retina			
^#^All retinal neurons	Not reported	*Chx10*	[40]
^!^All retinal neurons	Brain	*PrP*	[41]
Neural retina	Brian/multiple tissues	*Six3*	[42]
^#^All retinal neurons	Not reported	*Dkk3 *	[43]

*Expression with a tetracycline-inducible approach. ^!^Expression with a tamoxifen-inducible approach. ^#^Expression with BAC transgenic approach. ^$^Expression with knock-in approach. Abbreviations: *Chat*: choline acetyl transferase *Dct*: dopachrome tautomerase *Dkk3*: Dickkopf family protein 3 *Foxg1*: Forkhead box G1, *Glast*: glutamate/aspartate transporter, *Grik4*: glutamate receptor, ionotropic kainate 4 precursor, *hRgp*: human red/green pigment, *Math5*: murine atonal homolog 5, *mRho*: mouse rhodopsin, *mMo*: mouse *M-opsin*, *mSo*: Mouse S-opsin, *Opn4*: melanopsin, *Pcp2*: purkinje cell protein 2, *Pdgfra*: platelet-derived growth factor receptor-*α*, *PrP*: Prion protein, *Ptf1a*: pancreas specific transcription factor 1a, *Six3*: six/sine oculis subclass of homeobox gene, *Thy1.2*: Thymus cell antigen 1.2, and *Trp1*: tyrosinase-related protein.
